# Irritable Bowel Syndrome: Prevalence and Determinants Among Adults in the Makkah Region, Saudi Arabia

**DOI:** 10.7759/cureus.39568

**Published:** 2023-05-27

**Authors:** Tamara A Hafiz, Tala S Alhemayed, Renaad H Mandorah, Aeshah A Alshanqiti, Raneem A Almohaimeed, Osama M Noor

**Affiliations:** 1 Health Education & Health Promotion, Faculty of Public Health & Health Informatics, Umm Al-Qura University, Makkah, SAU; 2 Family Medicine, Faculty of Public Health & Health Informatics, Umm Al-Qura University, Makkah, SAU

**Keywords:** irritable bowel syndrome, prevalence, risk factors, stress, syndrome, ibs

## Abstract

Background: Irritable bowel syndrome (IBS) is among the most prevalent gut-brain interaction disorders and one of the most expensive in terms of money and health. Despite their widespread occurrence in society, these disorders have only recently been subjected to rigorous scientific inquiry, classification, and treatment. Although IBS does not lead to future complications, such as bowel cancer, it can impact work productivity and health-related quality of life and increase medical costs. Both young and older people with IBS have worse general health than the general population.

Aims: To determine the prevalence of IBS among adults aged 25 to 55 years in the Makkah region, as well as the risk factors that may contribute to it.

Methodology: A cross-sectional web-based survey with a representative sample (n = 936) of individuals in the Makkah region was carried out from November 21, 2022, to May 3, 2023.

Results: In Makkah, 420 out of 936 persons have IBS, making it 44.9% common. Most of the IBS patients in the study were women, aged 25 to 35 years, married, and suffering from mixed IBS. Age, gender, marital status, and occupation were found to be associated with IBS. It was discovered that there is an association between IBS and insomnia, medication use, food allergies, chronic diseases, anemia, arthritis, gastrointestinal surgery, and a family history of IBS.

Conclusion: The study highlights the importance of addressing the risk factors of IBS and developing supportive environments to alleviate its effects in Makkah. The researchers hope the findings inspire further research and action to improve the lives of people with IBS.

## Introduction

Irritable bowel syndrome (IBS) is one of the most common chronic functional gastrointestinal disorders (FGIDs). It is now defined as a disorder of gut-brain interaction with high financial and health costs. Despite its prevalence in society, such disorders have only recently been scientifically researched, classified, and treated based on well-designed clinical investigative studies [1]. IBS is characterized by abdominal pain or discomfort accompanied by changes in bowel habits and stool appearance without abnormalities in biochemical or tissue structure [2]. This condition is usually not progressive and does not lead to future complications such as bowel cancer, ulceration of the mucous membrane of the gastrointestinal tract (GIT), or even inflammation [3-5]. However, IBS may impact work productivity and health-related quality of life (QOL) and increase already rising medical costs [5]. IBS patients have been proven to have a significantly lower QOL [6,7]. IBS is the most prevalent digestive ailment to be diagnosed and the main reason people consult gastroenterologists [8]. It is estimated to affect 11% of the world's population [9], and one in five persons at some point in their lives [10]. According to the clinical presentation, IBS is further classified into sub-entities as follows: diarrhea-predominant IBS (IBS-D), constipation-predominant IBS (IBS-C), mixed (constipation/diarrhea) IBS (IBS-M) [11,12], and any unsubtyped group of IBS (IBS-U) [13].

The etiology of IBS has not yet been proven or identified by known causes. However, several risk factors related to it, such as sociodemographic factors, lifestyle, chronic conditions, and psychosocial factors [8,14], have been attributed to its pathogenesis. Additionally, circumstances associated with IBS, such as aberrant gut motor and sensory activity, central nervous system dysfunction, and other psychological disorders, have also been identified as contributing factors [15]. Studies that have detailed the frequency of IBS among countries are scarce; however, it may occur in Europe and North America at a rate of 10-15%, and much more frequently in the Asia-Pacific area (14% in Pakistan and 22.1% in Taiwan) [16,17]. The prevalence of IBS among the elderly may reach 31.8-40.7% [18,19], although this varies depending on gender and age group [20]. Females have been found to be about 1.5-3 times more likely than males to have IBS [21-24]. The incidence and prevalence of IBS are nearly equal in adults and adolescents between the ages of 15 and 65 years [25-27]. In addition, IBS cannot be diagnosed with diagnostic investigations or biomarkers [28]; it can be diagnosed only through clinical trials or by excluding other diseases [29]. There is no cure for IBS, and most treatments are symptomatic [30].

Saudi Arabian studies have shown that IBS accounts for 12% of primary healthcare visits and 28% of referrals to gastroenterologists [31,32]. Among these patients, only 15% seek medical attention [33]. Similar to severe chronic conditions like congestive heart failure, hepatic cirrhosis, renal insufficiency, and diabetes, IBS significantly impairs QOL [34,35]. Due to the lack of a commonly used validated symptom score, the comparison of studies evaluating the management of IBS has been hampered [36]. IBS' effects on people's functionality and QOL have been understated and neglected. Both young and older people with IBS have generally been reported to have worse general health than the general population. Patients with IBS appear to have a lower QOL in terms of their health than patients with specific other conditions [37].

To the best of the authors' knowledge, IBS has previously been studied among medical students and interns in the Jeddah area [38], and among nurses at King Abdulaziz University Hospital [39]. Additionally, there have been other studies in the northern Saudi Arabian province of Al-Jouf [13,20], as well as one conducted in the Majmaah region in 2017 among male students at Majmaah University [13]. The previous studies focused on the student/male segment, despite many studies indicating that women are more affected [21-24], and some studies compared it to a previous study in which there was a temporal and standard difference. Therefore, there is a lack of IBS studies that have been done for the general populace in Saudi Arabia's Makkah Al-Mukarramah region, and this is an addition to the world of research. This study aims to measure the prevalence and severity of IBS among adult patients from 25 to 55 years of age in the Makkah region and investigate the potential role of covariates (sociodemographic, medical, lifestyle, nutritional characteristics, chronic conditions, and psychological stress) as determinants of risk for IBS.

## Materials and methods

The prevalence of IBS was assessed using a structured cross-sectional web-based survey to collect data. The study was conducted from November 21, 2022, to May 3, 2023, in the Makkah region, which is considered the holy capital of the Kingdom of Saudi Arabia and is located in the western region.

The required sample size was calculated using the Raosoft sample size calculator (Raosoft, Inc., Seattle, WA). The minimum required sample size was determined to be 385 based on the following conditions: the total adult population aged between 25 and 55 years in the Makkah region is 4,729,807 (according to the 55th issue of the Statistical Yearbook published by the General Authority for Statistics in mid-2019), with a 50% response distribution, a 95% confidence level, and a 5% margin of error. To avoid bias, the sample size was doubled to 770.

Selection criteria

The inclusion criteria for this study were individuals from the general population between the ages of 25 and 55 years residing in Makkah Al-Mukarramah. Individuals under the age of 25 and those over the age of 55 years who do not live in the Makkah Al-Mukarramah region were excluded.

Then, the participants from 25 to 55 years of age living in Makkah were confirmed to have met the criteria for a possible diagnosis of IBS. The American Gastroenterological Association listed the following as exclusion factors: history of gastrointestinal surgery, severe weight loss, blood in the stool, and night awakenings due to abdominal pain, anemia, joint pain, and fever. Participants who reported one or more of these symptoms were excluded from the study.

After applying the exclusion criteria, we obtained a representative sample of the general population aged between 25 and 55 years old in the Makkah region, Saudi Arabia, consisting of 936 participants (out of the initial 1241 respondents who participated in the survey).

Data collection tools

The data were collected through structured self-questionnaires prepared in Arabic, which take five to eight minutes to complete. The questionnaire includes three sections. The first section contains social and demographic characteristics such as age, gender, nationality, social status, occupation, education, income, and medical history. The second section contains the Rome IV criteria for the diagnosis of IBS, the Bristol Stool Chart, and the Kessler Psychological Distress Scale (K10). The last section is related to lifestyle and some common risk factors that may affect or be associated with IBS, such as stress, type of diet, physical exercise, smoking, caffeinated beverage habits, and the presence of chronic diseases.

Regarding the Rome IV criteria, IBS is defined as the development of symptoms at least six months prior to diagnosis based on recurrent abdominal pain or discomfort for at least three days per month over the preceding three months and related to two or more criteria related to defecation, altered stool frequency, or associated with a difference in the shape or appearance of the stool.

The Bristol Stool Chart is a tool used to assess how long a stool has spent in the bowel. It categorizes stool into seven types based on their appearance and texture, with type 1 indicating the longest time spent in the bowel and type 7 indicating the least time. A normal stool is usually classified as a type 3 or 4. The appearance of types 1 or 2 with types 5 or 6 is considered indicative of IBS-M, a subtype of IBS (Table 1).

**Table 1:** Bristol Stool Chart IBS: irritable bowel syndrome.

Reference	Description
Type 1 (constipation) – IBS-C	Separate hard lumps, like nuts (hard to pass)
Type 2 (constipation) – IBS-C	Sausage shaped but lumpy
Type 3 (normal)	Like a sausage but with cracks on the surface
Type 4 (normal)	Like a sausage or snake, smooth and soft
Type 5 (diarrhea) – IBS-D	Soft blobs with clear-cut edges (passed easily)
Type 6 (diarrhea) – IBS-D	Flu­ffy pieces with ragged edges, a mushy stool
Type 7 (diarrhea)	Watery, no solid pieces, entirely liquid

The Kessler Psychological Distress Scale (K10) is a global measure of distress that consists of 10 items assessing anxiety and depressive symptoms. The scale measures the person's condition over the past four weeks, and the results range from 10 to 50 points. A score of less than 20 is considered well, while a score of 20-24 is classified as a mild mental disorder. A score of 25-29 is classified as a moderate mental disorder, and a score of 30 or more is classified as a severe mental disorder.

Data management

Through statistical data analysis software, IBM SPSS version 22 (IBM Corp., Armonk, NY), the extracted data were reviewed and encoded. Where a descriptive analysis was performed in the form of frequency and percentage of all demographic data and elements of IBS and risk factors for IBS. The chi-squared test was used to assess any differences in frequencies of qualitative variables by calculating the p-value, in which a p-value less than 0.05 was considered statistically significant. Finally, the results of the study were presented through tables and graphs.

Ethical consideration

Ethical approval was obtained from the Bioethics Committee at Umm Al-Qura University with the reference number HAPO-02-K-012-2023-02-1468 on February 21, 2023. Participants also received an online cover letter containing all the study details and were asked to provide their informed consent before starting the online survey. The focus was on their voluntary participation and their right to withdraw at any time without the need for an explanation.

## Results

Out of 1241 participants, 936 adult participants from the Makkah region met the inclusion criteria for the study (respondent rate: 75.42%). With a mean age of 36.19 years and a standard deviation of 7.84, the participants' ages ranged from 25 to 55 years old, and the majority (57.3%) belonged to the 25-35 years age group. More than half (53.5%) of the participants were female, and 50.9% were married. Only 1.1% of people have completed their primary or intermediate education, 9.7% have completed postgraduate study, 24.3% are students, and 29.9% work for the government. A total of 301 cases (32.1%) had a pre-diagnosis of IBS (Table 2).

**Table 2:** General characteristics of the participants

Characteristics		N = 936	%
Age (years)	25-35 years	536	57.3
	36-45 years	241	25.7
	46-55 years	159	17.0
Gender	Male	435	46.5
	Female	501	53.5
Nationality	Saudi	842	90.0
	Non-Saudi	94	10.0
Marital status	Married	476	50.9
	Unmarried	460	49.1
Educational level	Primary school	10	1.1
	Middle school	25	2.7
	High school/diploma	192	20.5
	Bachelor's degree	618	66.0
	Postgraduate	91	9.7
Occupation	Governmental sector	280	29.9
	Private sector	198	21.2
	Retired	33	3.5
	Student	227	24.3
	Do not work	198	21.2
Medical history	Pre-diagnosis of irritable bowel syndrome	301	32.1
	Gastrointestinal surgery	62	6.6
	Extreme weight loss	76	8.1
	Anemia	213	22.8
	Arthritis	322	34.4

**Figure 1:**
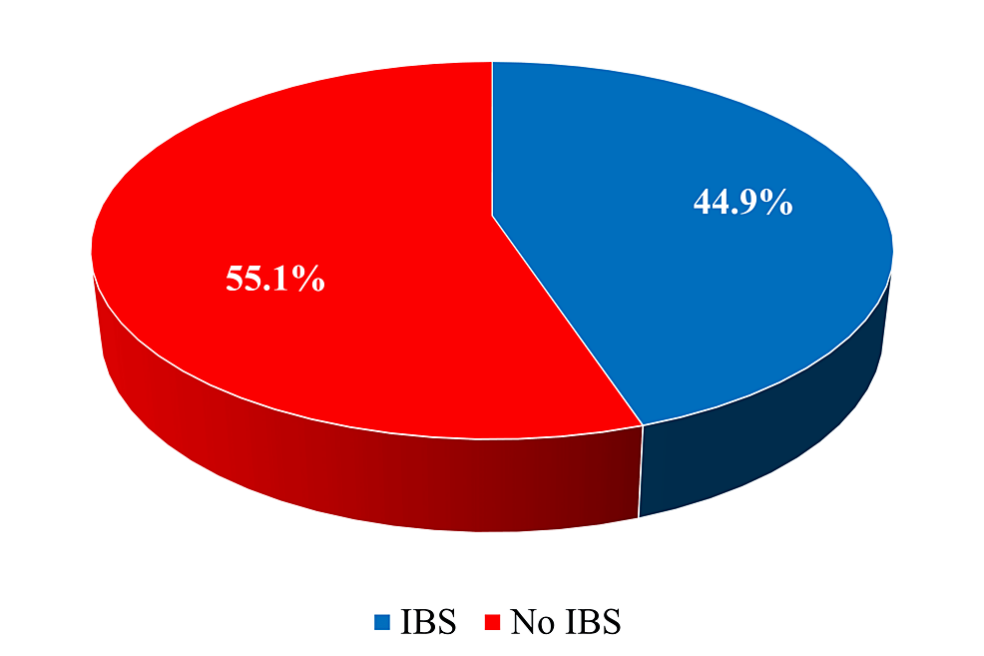
Prevalence of irritable bowel syndrome (IBS) among participants in Makkah, Saudi Arabia

Among 936 participants, 420 adults possibly with IBS, aged 25 to 55 years, were selected among the study participants, based on whether they met Rome IV criteria for a clinical diagnosis of IBS. Excluding other evident GIT issues. Of the 420 or 44.9% of participants diagnosed with IBS, 301 had previously been diagnosed and 119 had not been previously diagnosed (did not know about their condition) (Figure 1).

**Figure 2:**
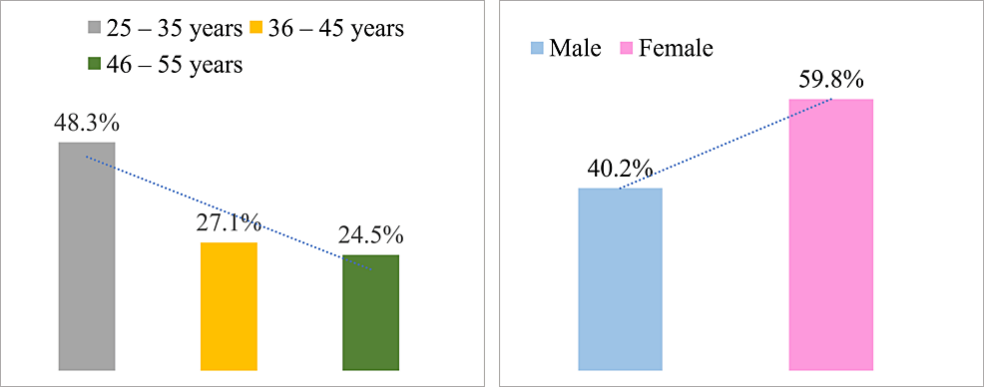
Distribution of irritable bowel syndrome among diagnosed participants in Makkah, Saudi Arabia (A) Distribution of irritable bowel syndrome according to age. (B) Distribution of irritable bowel syndrome according to gender.

IBS is distributed by age group in Figure 2A. According to the findings, the majority of participants with IBS (48.3%) were between the ages of 25 and 35 years, while the group with the lowest diagnosis was between the ages of 46 and 55 years (24.5%). In Figure 2B, IBS was less common in males (40.2%) compared to females (59.8%).

**Figure 3:**
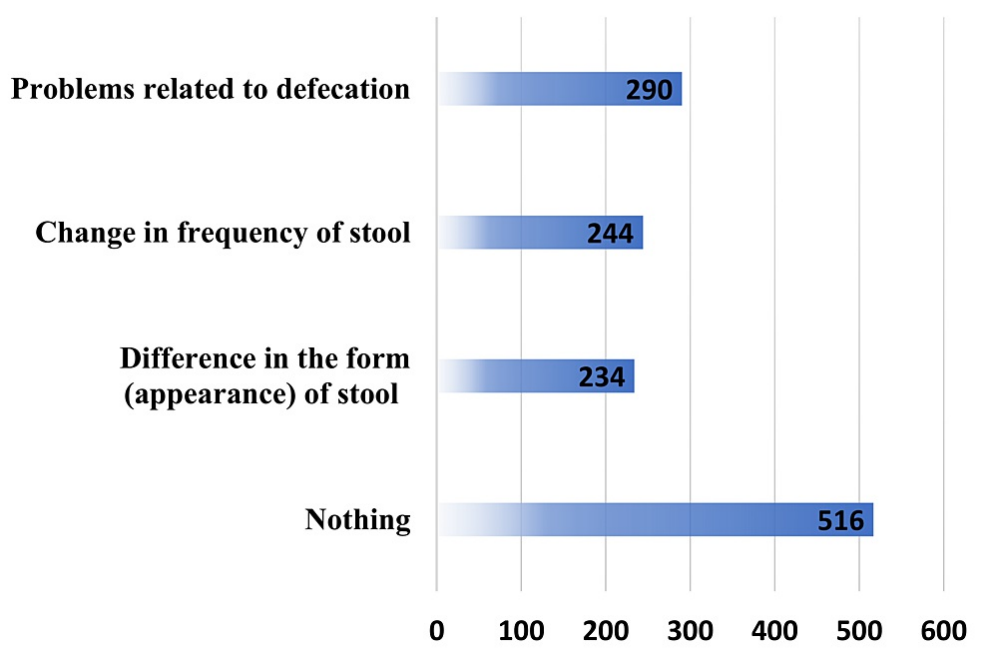
Participants' responses according to the Rome IV criteria for the diagnosis of irritable bowel syndrome

The results of the Rome IV criteria indicated that all symptoms related to defecation and stool, which were reported, were similar, with a slight difference, for the 420 participants diagnosed with IBS. On the other hand, no symptoms were reported for the 516 other participants (Figure 3).

**Figure 4:**
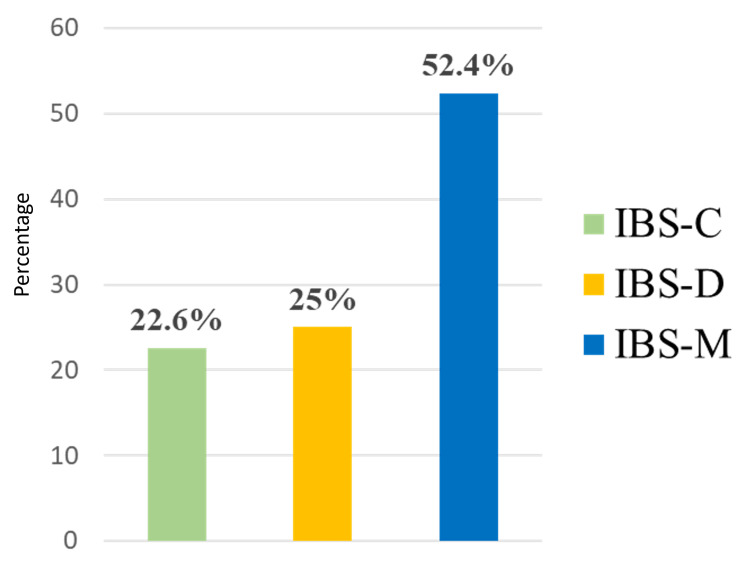
Classification of participants diagnosed with irritable bowel syndrome, according to the Bristol Stool Chart IBS-C: constipation-predominant irritable bowel syndrome; IBS-D: diarrhea-predominant irritable bowel syndrome; IBS-M: mixed (constipation/diarrhea) irritable bowel syndrome.

Of the 420 participants who had IBS, 220 had IBS-mix type (IBS-M), 105 had IBS-diarrhea type (IBS-D), and 95 had IBS-constipation type (IBS-C) (Figure 4).

When investigating the association between IBS and demographic factors, it was found that age and IBS are associated. The findings revealed that 203 of the participants aged 25-35 years had been diagnosed with IBS, as opposed to 103 participants aged 45-36 years with IBS (p = <0.001). The data also revealed an association between gender, marital status, and occupation (p = <0.001). According to the findings, women were diagnosed with IBS at a higher rate than men, who made up 251 (26.8%) of the total participants. Furthermore, the number of married people with IBS was 254, which is a larger segment than the unmarried segment, and the prevalence of IBS among government workers was higher than among others. In contrast, there was no association between an individual's education level or nationality and the prevalence of IBS (Table 3).

**Table 3:** Association of irritable bowel syndrome, based on Rome IV criteria scores, with demographic characteristics of participants * Significant.

Characteristics		Irritable bowel syndrome		P-value
		Yes	No	
Age (years)	25-35 years	203 (21.7)	333 (35.6)	0.000*
	36-45 years	114 (12.2)	127 (13.6)	
	46-55 years	103 (11.0)	56 (6.0)	
Gender	Male	169 (18.1)	266 (28.4)	0.001*
	Female	251 (26.8)	250 (26.7)	
Nationality	Saudi	381 (40.7)	461 (49.3)	0.487
	Non-Saudi	39 (4.2)	55 (5.9)	
Marital status	Married	254 (27.1)	222 (23.7)	0.000*
	Unmarried	166 (17.7)	294 (31.4)	
Educational level	Primary school	6 (0.6)	4 (0.4)	0.088
	Middle school	17 (1.8)	8 (0.9)	
	High school/diploma	87 (9.3)	105 (11.2)	
	Bachelor's degree	265 (28.3)	353 (37.7)	
	Postgraduate	45 (4.8)	46 (4.9)	
Occupation	Governmental sector	130 (13.9)	150 (16.0)	0.000*
	Private sector	89 (9.5)	109 (11.6)	
	Retired	20 (2.1)	13 (1.4)	
	Student	75 (8.0)	152 (16.2)	
	Do not work	106 (11.3)	92 (9.8)	
Medical history	Gastrointestinal surgery	39 (4.2)	23 (2.5)	0.003*
	Extreme weight loss	37 (4.0)	39 (4.2)	0.486
	Anemia	111 (11.9)	102 (10.9)	0.016*
	Arthritis	179 (19.1)	143 (15.3)	0.000*

Table 4 shows that according to the results of the Kessler Psychological Distress Scale (K10) used to assess participants' levels of psychological distress, 150 (37.4%) of the total population with the "well" condition were diagnosed with IBS, compared to 251 (62.6%) of those with a "well" condition and no diagnosis of IBS. On the other hand, out of a total of 190 people who were suffering from a "severe mental disorder," 113 (59.5%) were diagnosed with IBS, compared to 77 (40.5%) subjects with a severe mental disorder who had not been diagnosed with IBS. With a p-value of 0.000, this disparity indicates a statistically significant association between IBS and psychological distress.

**Table 4:** Association of irritable bowel syndrome, based on Rome IV criteria scores, with stress * Significant.

Kessler (stress)	Irritable bowel syndrome		P-value
	Yes	No	
Well	150 (16.0)	251 (26.8)	0.000*
Mild mental disorder	90 (9.6)	116 (12.4)	
Moderate mental disorder	67 (7.2)	72 (7.7)	
Severe mental disorder	113 (12.1)	77 (8.2)	

When investigating the association between IBS and medical characteristics, lifestyle, and nutrition as risk factors that may influence or be associated with IBS, Table 5 showed that there is an association between IBS and various medical characteristics, namely, family history (26.1%), chronic health problems (11.5%), history of gastroenteritis (14.6%), regular medication use (15%), insomnia (19.3%), and body mass index (19.9%). Food sensitivity (8%) is the least associated factor with IBS compared to the other associated factors.

On the other hand, there was no association between IBS, history of pelvic surgery (3.2%), smoking (10.6%), caffeine intake (29.7%), exercise (16.3%), and food source (Table 5).

**Table 5:** Association of irritable bowel syndrome based on Rome IV criteria scores between medical, lifestyle, and nutritional characteristics of participants * Significant.

Characteristics		Irritable bowel syndrome		P-value
		Yes, N (%)	No, N (%)	
Family history of irritable bowel syndrome	Yes	244 (26.1)	178 (19.0)	0.000*
	No	176 (18.8)	338 (36.1)	
Chronic health problem	Yes	108 (11.5)	87 (9.3)	0.001*
	No	312 (33.3)	429 (45.8)	
History of gastroenteritis	Yes	137 (14.6)	72 (7.7)	0.000*
	No	283 (30.2)	444 (47.4)	
History of pelvic surgery	Yes	30 (3.2)	31 (3.3)	0.484
	No	390 (41.7)	485 (51.8)	
Take medications regularly	Yes	140 (15.0)	78 (8.3)	0.000*
	No	280 (29.9)	438 (46.8)	
Insomnia	Yes	181 (19.3)	162 (17.3)	0.000*
	No	239 (25.5)	354 (37.8)	
Smoking	Yes	99 (10.6)	140 (15.0)	0.214
	No	321 (34.3)	376 (40.2)	
Caffeine intake	Yes	278 (29.7)	327 (34.9)	0.370
	No	142 (15.2)	189 (20.2)	
Exercise	Yes	153 (16.3)	219 (23.4)	0.062
	No	267 (28.5)	297 (31.7)	
Food hypersensitivity	Yes	75 (8.0)	66 (7.1)	0.031*
	No	345 (36.9)	450 (48.1)	
Food source	Homemade food	295 (31.5)	352 (37.6)	0.506
	Fast food	125 (13.4)	164 (17.5)	
Body mass index (kg/m^2^)	Normal (BMI = 18.5 to 24.9)	186 (19.9)	291 (31.1)	0.000*
	Overweight (BMI = 25 to 29.9)	160 (17.1)	174 (18.6)	
	Obese (BMI = 30 and over)	74 (7.9)	51 (5.4)	

## Discussion

This study offered a chance to assess the prevalence of IBS and look at the association between IBS and sociodemographic and health-related characteristics in a segment of adults in the Makkah region. The prevalence of IBS is high worldwide, albeit it varies depending on the nation and the diagnostic criteria applied [40]. IBS affects adolescents and adults at a rate of 10-20% in studies conducted in the West. In contrast, the frequency was not very high in Asian nations [41]. IBS is prevalent in Asian studies, affecting between 6.6% and 5.7% of the population, with the highest rates among people in their 20s [42,43]. According to the Rome IV criteria, the prevalence of IBS in Saudi Arabia was 18.2% [44] and 17.5% in the general population [45], 16.3% in medical doctors [46], and 15.8% in undergraduate students [47], while in medical students and interns at King Saud bin Abdulaziz University in the Jeddah region, it was 15.64% [38].

According to the findings of our study, 420 out of the 936 individuals, or 44.8% of the sample population, were diagnosed with IBS in the Makkah region. Our results show a higher prevalence of IBS in the Makkah region, in comparison to the past surveys conducted (2020) among male medical students at Majmaah University, Saudi Arabia, which show a lower IBS prevalence equal to 12.6% [13]. Another previous study had a prevalence of 31.8% among medical students and interns in Jeddah [19,48] and a 21% IBS prevalence was reported at King Saud bin Abdulaziz University for Health Sciences, Saudi Arabia among medical students [49].

When examining previous studies on the prevalence of IBS and the most prevalent subtypes, it was found in a study by Alharbi and colleagues that IBS-M is the most common subtype, followed by IBS-C, IBS-D, and IBS-U [50]. These findings are consistent with the findings of our current study, where the results revealed that the most common subtype is IBS-M (mixed type), followed by IBS-D (diarrhea) and IBS-C (constipation). It is also consistent with the results of a study conducted in Saudi Arabia [44]. For the latest research conducted in the Makkah region in 2013, the prevalence of IBS was as follows: IBS-M was the most common subtype, but IBS-C and IBS-D were equal in prevalence [51].

Upon analyzing the demographic characteristics of the participants, the researchers in this study found that females experience IBS more frequently than males. Additionally, people between the ages of 25 and 35 years, married individuals, those who work in the government sector or are unemployed, and those who have a family history of IBS also experience the condition more frequently compared to others. These findings are consistent with a previous study conducted in Saudi Arabia in 2019, which showed that IBS is most common in people between the ages of 20 and 40 years [52]. Furthermore, several reports have indicated that females have a higher incidence of IBS compared to males [19,21,53,54]. Genetic influence could also play a role in the development of IBS in 30% of people, as suggested by some reports [55,56].

In this study, the researchers uncovered a link between IBS and stress when they used the Kessler Psychological Distress Scale (K10) to investigate the association between the two. The study reveals that a higher proportion of individuals with a "Well" condition were not diagnosed with IBS, whereas among those with severe mental disorders, a greater number of individuals with IBS were identified than those without IBS. This disparity indicates a statistically significant association between IBS and psychological distress. Therefore, it can be inferred that a person's probability of having IBS increases with their level of psychological distress. These results are consistent with studies conducted in 2017 [38] and 2019 [44].

Furthermore, the researchers in this study found a significant association between the occurrence of IBS and a family history of IBS, the presence of chronic diseases, regular medication use, insomnia, food hypersensitivity, and body mass index. The study revealed that participants who were not diagnosed with IBS had a normal body mass index, whereas those diagnosed with the condition had a higher body mass index indicating obesity. In other words, the study found that individuals diagnosed with IBS were more likely to have a higher body mass index than those without the condition. This highlights the potential link between IBS and obesity, which may have important implications for both the prevention and management of this common gastrointestinal disorder. Similar results with the occurrence of IBS and a family history of IBS, the presence of chronic diseases, regular medication use, insomnia, food hypersensitivity, and body mass index were found in other studies [5,39]. However, a previous study contradicted these results by showing that there is no association between IBS, family history of IBS, and medication use [13].

In contrast, the present study did not identify an association between IBS and a history of pelvic surgery, smoking, caffeine intake, exercise, or food source. These findings are consistent with previous studies [13,39], which also reported no significant associations between IBS and these factors. However, a previous study contradicted these results by finding an association between IBS and smoking and exercise [47]. These conflicting findings suggest that the relationship between IBS and these factors is complex and may require further investigation to fully understand.

While the majority of earlier studies were in consensus that there is no conclusive evidence associating smoking with IBS, it is notable that an earlier study carried out in Saudi Arabia in 2019 produced inconsistent findings [44]. Similarly, research carried out by Jeddah medical students asserted an association between exercise and IBS [38]. Interestingly, previous studies have shown different results, even though our recent study found a significant association between IBS and a family history of the condition, gastroenteritis, food allergy, and body mass index. Further research is advised to confirm these associations with a larger population.

The strength of our study is that it sheds light on a crucial health issue that has implications for both individuals and society as a whole across a significant portion of Makkah's population by using a certified online questionnaire to ensure privacy and confidentiality. Moreover, the sample size was sufficient to give the study some power, excluding people over 55 and under 25, and who do not meet the American Gastroenterological Association's criteria. However, this study also has some limitations. First, it was voluntary, which could contribute to selection bias. Second, it was self-administered, which could result in inaccurate self-reporting. Finally, evaluating the chronology is problematic with a cross-sectional design.

## Conclusions

Our study found a substantial increase in the prevalence of IBS in Makkah compared to earlier studies, with the majority of people having IBS-M. The researchers in this study also identified several nutritional, medical, and demographic variables associated with IBS risk, as well as an association between stress and IBS. These findings have important implications for healthcare providers and health educators in Makkah, who should be aware of the high prevalence of IBS and the associated risk factors. They should also focus on developing supportive environments to alleviate the psychological and physical effects of IBS. Further research is needed to confirm our findings and explore the underlying mechanisms of the associations we identified. Longitudinal studies with larger sample sizes are needed to better understand the trajectory of IBS and its risk factors over time. The researchers hope the findings inspire further research and action to improve the lives of people with IBS in Makkah.
